# Non-Toxic Tau Oligomer Polymorphs Isolated from​ Non-Demented Individuals with Alzheimer’s Neuropathology

**DOI:** 10.1093/geroni/igaf122.3981

**Published:** 2025-12-31

**Authors:** Thai Nguyen, Jutatip Guptarak, Batbayar Tumurbaatar, Michela Marcatti, Wen-Ru Zhang, Anna Fracassi, Giulio Taglialatela

**Affiliations:** John Sealy School of Medicine, Galveston, Texas, United States; The University of Texas Medical Branch, Galveston, Texas, United States; The University of Texas Medical Branch, Galveston, Texas, United States; The University of Texas Medical Branch, Galveston, Texas, United States; The University of Texas Medical Branch, Galveston, Texas, United States; The University of Texas Medical Branch, Galveston, Texas, United States; The University of Texas Medical Branch, Galveston, Texas, United States

## Abstract

**Background:**

Alzheimer’s disease (AD) is a neurodegenerative disorder that is characterized by the presence of extracellular Aβ plaques and intracellular tau neurofibrillary tangles. A subset of individuals classified as A+T+N–, termed Non-Demented with Alzheimer’s Neuropathology (NDAN), maintain normal cognition despite harboring full AD pathology. This suggests that tau oligomers (TauO) may exist as distinct polymorphs with divergent functional consequences as well as the existence of innate protective mechanisms opposing the events that normally lead to cognitive impairment.

**Methods:**

We investigated whether NDAN brain-derived tau oligomers (NDAN-BDTOs) differ from AD-BDTOs in toxicity and microglial responses. Wild-type mice received intracerebroventricular injections of AD- or NDAN-BDTOs. Brain tissue was analyzed by immunofluorescence and western blotting to characterize microglial phenotypes (Iba1, CD68, TREM2) and assess pathways associated with phagocytosis, autophagy (Atg5, Atg7, LC3A/B, p62), and oxidative stress (SOD1/2, catalase, 8-oxo-dG).

**Results:**

AD- and NDAN-BDTOs were identified as structurally distinct polymorphs with divergent biological effects. AD-BDTOs induced neuronal apoptosis and pro-inflammatory microglial activation, while NDAN-BDTOs enhanced autophagy, preserved antioxidant defenses, and reduced oxidative DNA damage. Furthermore, NDAN-BDTOs elicited a specialized microglial phenotype distinct from that induced by AD-BDTOs, suggesting differential engagement of neuroinflammatory pathways.

**Conclusion:**

Our findings support the existence of a non-toxic, propagating tau oligomer in NDAN brains that promotes resilience by avoiding neurodegeneration. These results highlight the therapeutic potential of strategies that stabilize or redirect tau oligomers into non-toxic conformations, offering a novel avenue for disease-modifying interventions in AD.

